# Malaria in Russia

**Published:** 1897-11

**Authors:** 


Malaria in Russia.—Our St. Petersburg exchanges report the prevalence in some of the valleys and along the sluggish streams of Asiatic Russia of a malaria so malignant in its character and so rapid in its course as to amount almost to a pestilence. Deaths are numbered by the thousands, and the Russian garrison at Merv has been transferred to the Caspian Sea to escape annihilation. The attacks are somewhat similar to what has been seen in times past in peculiar locations along the low lands and bayous of the great rivers of the West, where the algid attack was so severe as to end in death.—*The Medical Times.*

